# Effects of Maternal Deprivation and Complex Housing on Rat Social Behavior in Adolescence and Adulthood

**DOI:** 10.3389/fnbeh.2018.00193

**Published:** 2018-09-11

**Authors:** Jiska Kentrop, Claire R. Smid, E. J. M. Achterberg, Marinus H. van IJzendoorn, Marian J. Bakermans-Kranenburg, Marian Joëls, Rixt van der Veen

**Affiliations:** ^1^Department of Translational Neuroscience, Brain Center Rudolf Magnus, University Medical Center Utrecht, Utrecht University, Utrecht, Netherlands; ^2^Department of Animals in Science and Society, Division of Behavioral Neuroscience, Faculty of Veterinary Medicine, Utrecht University, Utrecht, Netherlands; ^3^Department of Psychology, Education and Child Studies, Erasmus University Rotterdam, Rotterdam, Netherlands; ^4^Primary Care Unit, School of Clinical Medicine, University of Cambridge, Cambridge, United Kingdom; ^5^Faculty of Behavioural and Movement Sciences, Vrije Universiteit Amsterdam, Amsterdam, Netherlands; ^6^University Medical Center Groningen, University of Groningen, Groningen, Netherlands; ^7^Faculty of Social and Behavioural Sciences, Leiden University, Leiden, Netherlands

**Keywords:** early life stress, maternal deprivation, complex housing, environmental enrichment, social play, behavior, three-chamber social approach task

## Abstract

Early life context and stressful experiences are known to increase the risk of developing psychiatric disorders later in life, including disorders with deficits in the social domain. Our study aimed to investigate the influence of early life environment on social behavior in a well-controlled animal model. To this end we tested the effects of maternal deprivation (MD) on rat social play behavior in adolescence and social interaction in adulthood. Additionally, we provided a stimulating environment during adolescence (complex housing) as a potential intervention to diminish the effects of early life stress. Male and female Wistar rats were deprived from their mother for 24 h on postnatal day 3 (PND 3) or were left undisturbed. Complex housing started 5 days after weaning and consisted of housing 10 same-sex conspecifics in large, two-floor Marlau^TM^ cages until the end of the study. Social play behavior in adolescence was tested under different conditions (3 h vs. 24 h social isolation prior to testing). Maternally deprived males – but not females – showed a longer latency to play and a decreased total amount of social play behavior, after a 24 h isolation period. In adulthood, social discrimination was impaired in deprived male and female rats in the three-chamber social approach task. Complex housing did not moderate the effects of MD, but in itself induced a strong behavioral phenotype. Both complex housed males and females hardly displayed any play behavior after a 3 h isolation period. However, after 24 h of isolation, these animals showed shorter latencies to engage in social play behavior. Only complex housed males truly showed more social play behavior here, while showing less social interest in adulthood. We conclude that MD has mild negative effects on social behavior in adolescence and adulthood, which are not counteracted by complex housing. Complex housing induces a specific phenotype associated with rapid habituation; a lack of social play after short isolation periods, while increasing play behavior after a prolonged period of isolation in adolescence, and less social interest, paired with intact social discrimination in adulthood. In both early life settings, males seem to be more influenced by the early life environment compared to females.

## Introduction

Humans and many other animals critically depend on social interaction with conspecifics for survival. In order to thrive in society, it is essential to properly process social information and provide adequate responses in social contexts. Conversely, not being able to partake in society heavily compromises health and well-being, as seen in numerous psychiatric disorders such as depression and schizophrenia. An adverse early life environment is known to increase the risk of developing psychiatric disorders later in life, including disorders with deficits in the social domain (Dube et al., 2003; Carr et al., 2013). Understanding how the early life environment contributes to the development of dysfunctional social behavior may help to elucidate the mechanisms underlying psychopathology. Animal models allow investigation of this question under controlled conditions.

In rodents, the early postnatal environment is strongly determined by interactions of pups with the mother. Animal models of disrupted mother–pup interactions in the early postnatal period have provided valuable insights into the effects of early life stress (ELS) on behavior, with a prominent role of the hypothalamus–pituitary–adrenal (HPA) axis. Deprivation of maternal care leads to a surge in circulating corticosterone and HPA-axis activity in a period where HPA-axis activity is normally low. With this approach we aim to model cortisol overexposure caused by neglect or trauma in young children. Corticosterone overexposure caused by depriving rats or mice from maternal care once for 24 h, or repeatedly for 3 h per day in the first two postnatal weeks is known to alter HPA-axis functionality and stress reactivity later in life and quite consistently causes deficits in cognitive and emotional behavior (for reviews see Lupien et al., 2009; Krugers and Joëls, 2014; Marco et al., 2015; Chen and Baram, 2016; Tractenberg et al., 2016). However, less is known about the effects of ELS on social behavior.

Social play behavior, also referred to as play-fighting, is one of the most characteristic social behaviors seen in adolescent mammals, including rats and children, and one of the first social behaviors that does not include interactions with the mother. It is thought that social play behavior is a precursor of adult social, aggressive, and sexual behavior that is displayed out of context, in exaggerated form and functions to facilitate the development of circuits involved in social behavior (Panksepp, 1981; Vanderschuren and Trezza, 2014; Pellis and Pellis, 2017). Indeed, rats that were reared in social isolation during the period when play is most abundant show long-lasting social impairments that can be restored by 1 h of resocialization per day (Potegal and Einon, 1989). Alterations in adolescent social play behavior may be related to impaired development of brain areas such as the prefrontal cortex, striatum, and amygdala (Vanderschuren and Trezza, 2014). In rats, 3–6 h of daily maternal separation in the first 2 or 3 weeks of life either increased (Veenema and Neumann, 2009; Zimmerberg and Sageser, 2011; Lundberg et al., 2017), decreased (Muhammad and Kolb, 2011), or did not affect (Arnold and Siviy, 2002) play behavior. Recently, our lab showed a positive correlation between the amount of maternal care received in the first week of life and the amount of social play behavior in adolescent male but not female rats (Van Hasselt et al., 2012), indicating that maternal care is an important factor in the development of adolescent social behavior.

Effects of ELS on adult social behaviors have also been reported. ELS is known to increase aggression in rodents (Veenema et al., 2006, 2007; Zamberletti et al., 2012; Yu et al., 2013; Kohl et al., 2015), but effects on social interest and social discrimination are less consistent. It was observed that 3 h of daily maternal separation reduced social interest (i.e., time spent exploring a conspecific) in both adult male and female mandarin voles (Wei et al., 2013; Yu et al., 2013) and mice (Tsuda and Ogawa, 2012; Zoicas and Neumann, 2016). In rats, social interest was reduced in males – but not females – after 12 h MD on PND 9 and 11 (Takase et al., 2012), while 24 h MD on PND 9 did not affect male or female social exploration (Zamberletti et al., 2012). Social discrimination (or preference for social novelty; i.e., the ability to discriminate between a familiar and unfamiliar conspecific) was impaired after daily maternal separation in male rats (Lukas et al., 2011), but unaffected in male mice (Zoicas and Neumann, 2016).

Besides the early postnatal period, adolescence is gaining interest as a sensitive period in which environmental influences can impact brain development (Fuhrmann et al., 2015). In laboratory conditions, this is mostly studied by manipulating the way in which the animals are housed, either by providing an impoverished environment (isolation rearing), or a more complex, socially and/or physically enriched environment. Generally, rodents are either single housed or group housed ranging from two to four rats per cage, with bedding and free access to water and food. In enriched conditions rodents are housed in larger groups in enlarged cages equipped with physical enrichment such as tunnels, shelters, and/or running wheels. Efforts to standardize enriched environments over labs have led to cages that are socially, physically, and even cognitively challenging (Simpson and Kelly, 2011). In general, environmental enrichment has been shown to decrease anxiety and increase learning and memory (van Praag et al., 2000; Simpson and Kelly, 2011). We have previously found complex housing to increase attention, but diminish behavioral inhibition (van der Veen et al., 2015). More recently, beneficial effects of environmental enrichment on social behavior have been shown (Brenes et al., 2016; Kentner et al., 2018). With regard to ELS, environmental enrichment has been successfully applied as intervention strategy to diminish or reverse ELS-induced changes in stress reactivity (Francis et al., 2002), anxiety (Lukas et al., 2011), and emotional (Vivinetto et al., 2013) or spatial learning (Cui et al., 2006). Given these successful interventions and the beneficial effects of complex rearing environments on social behavior, complex housing could be a potential intervention strategy for ELS-induced impairments in social behavior as well.

The aim of this study was threefold. First, we investigated the effects of early maternal deprivation (MD) (24 h on PND 3) on rats’ social competence in adolescence and adulthood. Second, we examined the effects of a complex rearing environment (from PND 26 onward) on these same measures. Finally, we tested for interactive effects of the two manipulations, resulting in compensatory or additive effects. Social play behavior was observed during adolescence. In adulthood rats were tested in the three-chamber social approach task to measure social interest and social discrimination.

## Materials and Methods

### Animals

**FIGURE 1:**
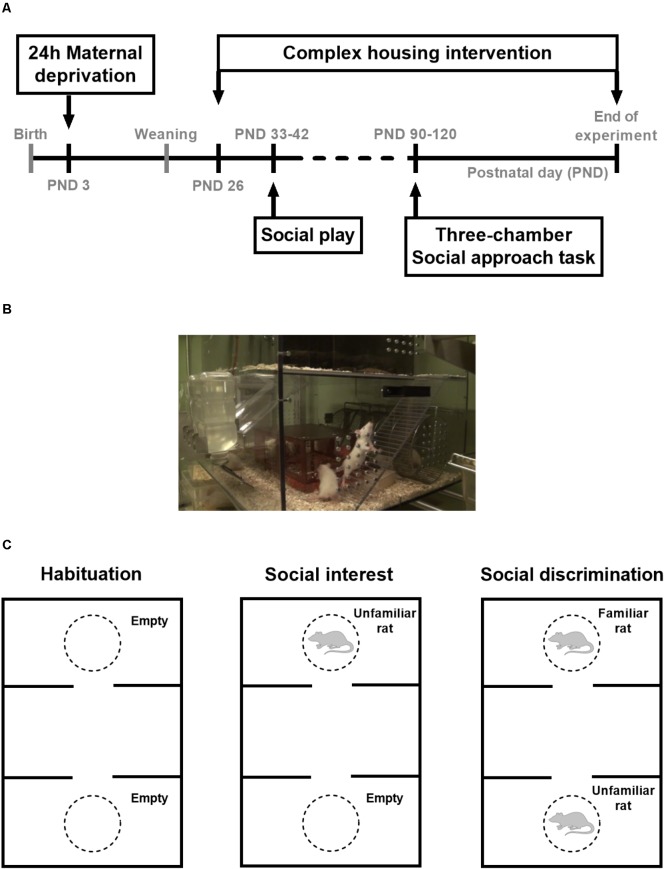
**(A)** Timeline of the experiments, **(B)** picture of the complex housing Marlau^TM^ cages for rats (manufactured by Viewpoint, France), and **(C)** Schematic representation of the three-chamber social approach task, containing a habituation, social interest, and social discrimination phase. During the social interest phase, an unfamiliar rat was placed in one of the cylinders, while the other cylinder remained empty. Rats are known to prefer exploring conspecifics over inanimate objects and this social preference was quantified with a discrimination index (DI) that indicates the percentage of time spent near the unfamiliar rat. During the social discrimination phase, the unfamiliar rat from the previous phase was now considered familiar and a new, unfamiliar rat was placed in the other cylinder. Given the choice between a familiar and unfamiliar rat, rats are expected to explore the unfamiliar stimulus rat. Social discrimination is represented with a DI that depicts the preference for the unfamiliar rat, i.e., the percentage of time spent near the unfamiliar rat.

Male and female Wistar rats were obtained at 8–10 weeks of age (Charles River Laboratories, Saint-Germain-Nuelles, France). Animals were kept in a temperature (21°C) and humidity (55%) controlled room with a 12 h light–dark cycle (lights on at 7:00 AM). Breeding started after animals had been familiarized with our animal facility for at least 2 weeks. Food and water were available *ad libitum*. The offspring of 63 dams, consisting of both males (*n* = 144) and females (*n* = 166) was tested on social behavior in adolescence and adulthood (see **Figure 1A** for a timeline). Data on adolescent social play behavior after 3 h social isolation, adult social approach behavior, and body weights were obtained from the same set of animals with *n* = 16 rats per group, unless otherwise specified. Data on social play behavior after 24 h social isolation come from follow-up experiments that were conducted with a separate set of animals (males: *n* = 10 couples per group and females: *n* = 12–13 couples per group). Adolescent testing took place when the animals were between 33 and 42 days of age, adult testing took place between 12 and 18 weeks of age. All tests took place in the morning between 8:00 and 13:00. In the adult three-chamber social approach task, 22 male and 44 female age-matched Wistar rats served as stimulus animals. These stimulus animals were socially housed in groups of two males or four females. Once per week cages were cleaned and general health status was checked. Cages were provided with a woodblock as standard cage enrichment. Experiments were approved by the local committee for Animal Health, Ethics and Research of Utrecht University. Animal care was conducted in accordance with the EC Council Directive of November 1986 (86/609/EEC).

### Early Life Experience: Breeding and Maternal Deprivation

Two females were paired with a male for 10 days. After separation from the male, females stayed together for another week and were then individually housed to prepare for birth. A paper towel was provided to the mothers as nesting material. At PND 3, dams were taken out of their home cage and placed in another cage. The sex of the pups was determined and when necessary litters were culled to a minimum of 6 (by cross-fostering) and a maximum of 10 pups. All litters contained at least two pups from each sex. Litters were randomly assigned to the MD or control condition. Mothers and pups in the control group were placed back into their home cage within 2 min, while for the experimental group MD started. During MD, litters stayed together in their home cage (without the dam, who remained single housed in a separate cage without pups, with food and water *ad libitum*) and were transported to a different room. The cage in which the pups were housed was placed on a heating pad (33°C) to prevent hypothermia of the pups. During MD, the pups were left undisturbed and they were not fed. After 24 h the cage was taken back to the original room and the mother was reunited with her litter. The week following deprivation, mothers from deprived litters were not provided with standard cage enrichment (paper tissue and woodblock).

### Early Adolescence: Weaning and Complex Housing Environment

Pups were weaned at 21 days of age and randomly assigned to the standard or complex housing condition with a maximum of two siblings per experimental group. From each litter, some pups were placed in the complex housing group and others in the control group (standard housing) to minimize litter effect. At weaning, all animals were housed in Makrolon cages (37 × 20 × 18 cm), in same-sex groups of three to four rats per cage, with all rats having the same early life background (either MD or control). Rats in standard housing stayed in this condition until social play testing; after social play testing was finished, they were pair-housed. Animals in the complex housing condition were transferred on PND 26 to Marlau^TM^ cages (Viewpoint, Lyon, France; **Figure 1B**), housing 10–11 same-sex animals per cage. Marlau cages (60 × 80 × 51 cm) have two floors and provide a complex and challenging environment for the rats (Fares et al., 2013). The first floor contains a big compartment with three running wheels, a shelter, *ad libitum* access to water, two woodblocks, and a climbing ladder to the second floor, where a maze has to be passed to gain access to a tube leading to the food compartment on the first floor. Via a one-way passage rats could regain access to the bigger first floor compartment. The maze was changed once per week (alternating between 12 different configurations), assuring novelty and sustained cognitive stimulation. Territorial dominance was avoided by the presence of two gates on each side of the maze. To prevent disturbance, mazes were not changed in the weeks of behavioral testing. A more detailed description of the experimental setup is given elsewhere (van der Veen et al., 2015).

### Adolescent Social Play Behavior

The social play setup consisted of a transparent acrylic arena (40 cm × 40 cm × 60 cm) with 2 cm of wood shavings on the floor. All social play experiments were performed under red light conditions. Rats were habituated to the test arena for 10 min together with cage mates on two different days in the week preceding testing. To increase motivation to play on the testing day, the animals were socially isolated for 3 (Experiment 1) or 24 h (Experiment 2) prior to testing. In a play session two animals were placed in the arena for a 15 min interaction period that was recorded with a camera for offline behavioral coding.

Social play behavior was coded by two trained observers who were blind to the experimental condition of the animals, using The Observer XT version 9.0 software (Noldus Information Technology B.V., Wageningen, Netherlands). Inter-rater reliability was determined by calculating intraclass correlations for all coded behaviors and ranged between 0.97 and 0.99. Behaviors that were observed included pouncing and pinning frequency, pin latency, pin length, social exploration, and social rest (i.e., huddling) duration. “Pouncing,” a measure for play initiation, is defined as one animal approaching and soliciting another, during which the soliciting rat attempts to nose or rub the nape of the neck of the play partner. In response to being pounced upon, the solicited rat can fully rotate to a supine position, which is defined as “pinning” (Panksepp et al., 1984; Vanderschuren et al., 1997). Pouncing and pinning are considered the two most characteristic play behaviors. Pouncing and pinning are highly correlated (Pearson’s *r* = 0.98, *p* < 0.001 in the current dataset) and therefore only pinning is reported in this study as this requires both animals to be engaged in play. Other, non-play related, social behaviors such as allo-grooming, licking, sniffing, and touching the other rat were coded as “social exploration.” “Social rest,” also referred to as huddling, occurs in an inactive period in which the two rats are lying still while maintaining physical contact. During social rest both rats are either inactive or sleeping, but not busy with social exploration or non-social exploration such as self-grooming or cage exploration.

To control for the potential effect of dominance on play, couples were age- and weight-matched (<10 g difference in body weight). Only couples were tested that had not previously encountered each other (i.e., no siblings or cage mates). Since social play behavior depends on the engagement of both animals, a play couple had the same experimental background and was considered as one experimental unit. Testing took place between 33 and 42 days of age, well within the period in which rats show abundant amounts of social play behavior (Panksepp, 1981).

### Adult Three-Chamber Social Approach Task

The three-chamber setup (120 cm × 80 cm × 40 cm) consisted of a black acrylic floor and transparent acrylic walls that separate the arena in three equal sized compartments (Sociability cage for rats, Noldus Information Technology B.V., Wageningen, Netherlands, see **Figure 1C** for a schematic representation). The walls of the inner compartment contained an opening to the outer compartments that could be closed with removable slide doors. Two cylinders with a diameter of 22 cm (40 cm in height) were placed in the outer compartments for the stimulus rats during testing. These cylinders were made of acrylic bars placed 15 mm apart to allow for social contact while preventing aggressive assaults. All three-chamber experiments were performed in dim light conditions (10 lux). Between tests, the arena and cylinders were cleaned with a 0.5% v/v solution of Shureclean VK 10 (JohnsonDiversey, United Kingdom) dissolved in warm water. In two 5-min habituation sessions in the week before testing animals (one at a time) had access to all compartments, without the cylinders being present. Unfamiliar, sex- and age-matched Wistar rats ordered from Charles River were used as stimulus animals. The stimulus animals were habituated twice for 5 min to the cylinders. A stimulus animal was used for a maximum of three tests per day.

The experimental procedure consisted of three phases (**Figure 1C**). In principle, all phases lasted 5 min and 5 min was used for the analyses. However, the social interest phase is prolonged to 10 min, to allow sufficient access time to the novel rat to be able to consider it familiar in the social discrimination phase. Between phases, the test rat was placed in the middle compartment for ca. 1 min, using the removable doors to block entrance to the outer compartments.

Phase I: Habituation with cylinders present (5 min): The rat was placed in the middle compartment and habituation started with removal of the slide doors that provided access to the outer compartments. The rat was allowed to freely explore all three compartments and the two cylinders that were placed in the outer compartments. In this phase the cylinders were empty, but unfamiliar and therefore represented novel objects. After habituation, the test rat was confined in the middle compartment for 1 min before the social interest phase started.

Phase II: Social interest (10 min): During the social interest phase, an unfamiliar stimulus rat was placed in one of the cylinders (counterbalanced across rats), while the other cylinder remained empty. Again, both doors were opened and the test rat had access to all compartments. The test rat could roam around for 10 min, to ensure sufficient time to explore both compartments and to ensure familiarization with the stimulus rat. Since the first 5 min is most informative in terms of exploration, only the first 5 min was used for data analysis. After the social interest phase, the test rat was confined in the middle compartment for 1 min before the social discrimination phase started.

Phase III: Social discrimination (5 min): The stimulus rat from phase II was now considered familiar and a different, unfamiliar stimulus rat was placed in the other cylinder. Both doors were opened and the test rat was allowed to explore the setup for 5 min.

As a prerequisite for data analysis, only animals that left the middle compartment during the habituation phase and animals that spent time with the unfamiliar rat during the social interest phase were included for data analysis. No animals had to be excluded based on these criteria. Five complex housed females (of which four no-MD and one MD) were excluded from the analysis because they jumped on top of the three-chamber set-up and as a consequence their Ethovision track could not be used for data analysis.

Ethovision XT version 9 was used to automatically track the rats and locate them in different zones of the arena (Noldus Information and Technology B.V., Wageningen, Netherlands). Next to three chamber zones, two cylinder zones were defined. The cylinder interaction zones refer to a 10 cm perimeter around the cylinders. Analyzed parameters included distance moved, latency to approach the cylinder zones, time spent in different rooms, and time spent in the cylinder zones. A discrimination index (DI) was calculated to quantify the preference of the test rat for one of the cylinders by calculating the percentage of total cylinder exploration time spent in a specific cylinder zone, using the formula:

$$\text{Discrimination index (DI)=}\left(\frac{\text{Time spent near cylinder}X}{\text{Time spent near cylinder }X+\text{cylinder }Y}\right)*100\%,$$

where *X* is the cylinder zone containing the unfamiliar stimulus rat (social interest phase) or new unfamiliar stimulus rat (social discrimination phase).

### Estrous Cycle

To account for the potential influence of sex hormones on adult social behavior in females, vaginal smears were collected 2–3 h after females were tested in the three-chamber social approach task. Vaginal smears were obtained by inserting the head of a sterile plastic smear loop (1 μL, VWR) and gently swabbing the vaginal wall. The collected cells were transferred to a drop of water on a glass microscope slide, air-dried, and stained with a 5% Giemsa (Sigma–Aldrich Chemie B.V., Zwijndrecht, Netherlands) in water solution. Microscopic evaluation of the cells present in the vaginal smears was used to determine the phase of the estrous cycle as a proxy for circulating levels of female sex hormones. Smears were assigned as proestrus, estrus, or metestrus–diestrus, according to Cora et al. (2015).

### Statistical Analyses

Statistical analyses were performed using SPSS for windows version 23 (IBM, United States). Results are presented as mean ± SEM. Outlying scores (3.29 standard deviations below or above the mean) were winsorized [substituted with the next highest score (Tabachnick and Fidell, 2006)] to mitigate excessive influence of outliers without excluding subjects. In total, 26 data points were winsorized in 18 different variables. As cross-fostering of pups on PND 3 could be a potential confounder (the presence or absence of), cross-fostering within a litter was included as a covariate in the statistical analyses of all behavioral parameters. Cross-fostering was not significantly associated with any of the parameters and thus not included in the final analyses. Also estrous cycle stage of the females as a covariate was not associated with the adult behavioral parameters and not included in the final analyses.

Two-way univariate ANOVAs were performed to compare group differences in body weight at the time of social play and at 12 weeks of age. Behavioral data were analyzed using two-way univariate ANOVAs or repeated measures ANOVAs where appropriate. Early life experience (no-MD vs. MD) and housing condition (standard housing vs. complex housing) served as between-subject factors in the two-way univariate ANOVAs and location or time served as repeated factor in the repeated measures ANOVAs. For the three-chamber social approach task, the DI of every group was analyzed with a one sample Student’s *t*-test against 50% chance level. A possible relation between time spent in proximity to the stimulus rat in the social interest phase and the DI for the social discrimination phase was tested using Pearson’s *r*. For future meta-analytical purposes, all statistics are added in **Supplementary Tables S1–S5**.

## Results

### Body Weight

#### Males

At the time of social play testing, maternally deprived males weighed less than non-deprived males [*F*(1,52) = 6.92, *p* < 0.05, ${\text{η}}_{\text{p}}^{2}$ = 0.12; **Table 1**, upper rows]. Moreover, complex housed males had a lower body weight compared to standard housed males [*F*(1,52) = 11.54, *p* < 0.001, ${\text{η}}_{\text{p}}^{2}$ = 0.27). Housing did not moderate the body weight effects of MD [MD^∗^housing *F*(1,52) = 0.00, *p* = 0.95, ${\text{η}}_{\text{p}}^{2}$ = 0.00]. At the time of adult testing (12 weeks of age), body weight was comparable across all groups [MD *F*(1,60) = 0.02, *p* = 0.88, ${\text{η}}_{\text{p}}^{2}$ = 0.00, housing *F*(1,60) = 1.64, *p* = 0.205, ${\text{η}}_{\text{p}}^{2}$ = 0.03, MD ^∗^ housing *F*(1,60) = 0.89, *p* = 0.35, ${\text{η}}_{\text{p}}^{2}$ = 0.02].

**Table 1:** Body weight (BW) in adolescence and adulthood in maternally deprived (MD) and complex housed male and female rats.

Variables		No-MD		MD	
		Standard housing	Complex housing	Standard housing	Complex housing
Males	Adolescent BW (g)	120.69 ± 2.66	111.71 ± 2.88^∗∗∗^	122.17 ± 5.81^$^	105.06 ± 2.37^$∗∗∗^
	Adult BW (g)	333.44 ± 7.55	331.75 ± 4.08	337.34 ± 3.75	326.31 ± 3.39
Females	Adolescent BW (g)	95.25 ± 2.47	100.06 ± 1.58	88.50 ± 1.22^$$$^	90.50 ± 1.47^$$$^
	Adult BW (g)	204.50 ± 2.95	191.63 ± 2.81^∗∗∗^	193.69 ± 2.49^$$^	186.69 ± 2.09^$$∗∗∗^

*Numbers represent mean ± SEM, ^$^*p* < 0.05, ^$$^*p* < 0.01, and ^$$$^*p* < 0.001 depict main effects of MD, ^∗∗∗^*p* < 0.001 depicts main effects of housing. There were no interaction effects. Group sizes *n* = 16 for all measures except adolescent no-MD complex housing males *n* = 10 and adolescent MD standard housing males *n* = 14, due to missing values*.

#### Females

Like males, at the time of social play testing maternally deprived females weighed less than non-deprived controls [*F*(1,60) = 21.75, *p* < 0.001, ${\text{η}}_{\text{p}}^{2}$ = 0.27; **Table 1**, lower rows]. Complex housing did not significantly influence adolescent body weight in females [*F*(1,60) = 3.79, *p* = 0.056, ${\text{η}}_{\text{p}}^{2}$ = 0.06], nor did it moderate the effect of MD [MD ^∗^ housing *F*(1,60) = 0.65, *p* = 0.42, ${\text{η}}_{\text{p}}^{2}$ = 0.01]. At the time of adult testing, maternally deprived females still weighed less than non-deprived females [*F*(1,60) = 9.14, *p* < 0.01, ${\text{η}}_{\text{p}}^{2}$ = 0.13]. In addition, adult complex housed females weighed less than their standard housed controls [*F*(1,60) = 14.56, *p* < 0.001, ${\text{η}}_{\text{p}}^{2}$ = 0.20]. Complex housing did not moderate MD effects [MD ^∗^ housing *F*(1,60) = 1.27, *p* = 0.26, ${\text{η}}_{\text{p}}^{2}$ = 0.02].

### Adolescent Social Play Behavior

#### Social Play Behavior Following 3 h of Social Isolation in Males and Females

After 3 h of social isolation, none of the play behaviors were affected by MD, nor did MD interact with the effects of complex housing in males or females (**Table 2**). However, both male and female complex housed animals showed minimal amounts of social play behavior compared to standard housed rats [number of pins males: *F*(1,28) = 23.22, *p* < 0.001, ${\text{η}}_{\text{p}}^{2}$ = 0.45, females: *F*(1,26) = 28.11, *p* < 0.001, ${\text{η}}_{\text{p}}^{2}$ = 0.52]. Time spent in social exploration was unaffected by housing condition in both males and females (**Supplementary Table S1**). In contrast to social play behavior, we observed social resting behavior in the complex housed animals which was absent in standard housed males [*F*(1,28) = 16.93, *p* < 0.001, ${\text{η}}_{\text{p}}^{2}$ = 0.38] and females [*F*(1,26) = 8.41, *p* < 0.01, ${\text{η}}_{\text{p}}^{2}$ = 0.24].

**Table 2:** Adolescent social play behavior after 3 h of social isolation in maternally deprived (MD) and complex housed male and female rats.

Variables		No-MD		MD	
		Standard housing	Complex housing	Standard housing	Complex housing
Males	Amount of pins (#)	16.50 @ 4.11	3.00 @ 1.30***	16.88 @ 3.75	2.38 @ 1.05***
	Social exploration (s)	227.20 @ 16.91	270.09 @ 28.00	267.89 @ 29.85	265.63 @ 31.26
	Social rest (s)	0.00 @ 0.00	33.43 @ 16.76***	0.00 @ 0.00	90.16 @ 24.93***
Females	Amount of pins (#)	16.25 @ 3.50	0.00 @ 0.00***	14.43 @ 4.73	0.00 @ 0.00***
	Social exploration (s)	253.59 @ 18.94	312.48 @ 50.32	276.01 @ 11.70	297.58 @ 20.64
	Social rest (s)	0.00 @ 0.00	30.32 @ 19.42**	0.00 @ 0.00	85.89 @ 33.29**

*Numbers represent mean ± SEM, ^∗∗^*p* < 0.01 and ^∗∗∗^*p* < 0.001 depict main effects of housing. There were no effects of MD and no interaction effects. Group sizes males: *n* = 8 couples for all groups, females: *n* = 8 no-MD standard housing, *n* = 7 no-MD complex housing, *n* = 7 MD standard housing, *n* = 8 MD complex housing*.

#### Social Play Behavior Following 24 h of Social Isolation

Although the strong reduction of social play behavior after 3 h of social isolation in both complex housed males and females provides valuable insights in the impact of housing conditions, it does not allow for a quantitative analysis of social play behavior. Social isolation prior to testing increases the motivation for social play behavior and this reaches a maximum after 24 h of social isolation (Panksepp and Beatty, 1980). In order to gain insight in the possible effects of housing condition on social play behavior, the experiment was repeated with a different set of animals where a social isolation period of 24 h before testing was used. In this experiment, none of the males or females showed social resting behavior.

#### Males

**FIGURE 2:**
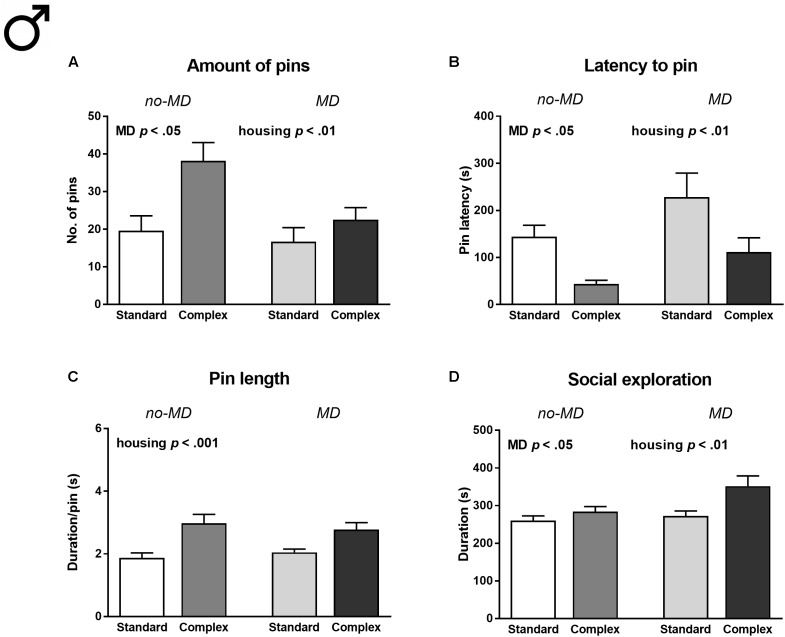
Male adolescent social play behavior following 24 h of social isolation in maternally deprived (MD) and complex housed rats. **(A)** Total amount of pins, **(B)** latency to pin, **(C)** pin length, and **(D)** time spent in social exploration. Statistics depict main effects. Graphs represent mean + SEM. *n* = 10 play couples per experimental group.

In male animals, MD was associated with a decreased amount of social play [number of pins *F*(1,36) = 5.49, *p* < 0.05, ${\text{η}}_{\text{p}}^{2}$ = 0.13, **Figure 2A**], increased latency to engage in social play [latency to pin *F*(1,36) = 5.65, *p* < 0.05, ${\text{η}}_{\text{p}}^{2}$ = 0.14, **Figure 2B**], and increased social exploration [*F*(1,36) = 5.13, *p* < 0.05, ${\text{η}}_{\text{p}}^{2}$ = 0.13, **Figure 2D**]. Contrary to the reduction in social play seen after 3 h of isolation, complex housed males showed a significantly higher amount of social play behavior compared to standard housed males after 24 h of isolation [*F*(1,36) = 9.44, *p* < 0.01, ${\text{η}}_{\text{p}}^{2}$ = 0.21; **Figure 2A**]. Moreover, complex housed males showed a reduced latency to play [*F*(1,36) = 11.77, *p* < 0.01, ${\text{η}}_{\text{p}}^{2}$ = 0.25; **Figure 2B**], increased pin length [*F*(1,36) = 20.67, *p* < 0.001, ${\text{η}}_{\text{p}}^{2}$ = 0.37; **Figure 2C**], and an increase in social exploration [*F*(1,36) = 8.64, *p* < 0.01, ${\text{η}}_{\text{p}}^{2}$ = 0.19; **Figure 2D**]. Complex housing did not moderate the effects of MD (**Supplementary Table S2**).

#### Females

**FIGURE 3:**
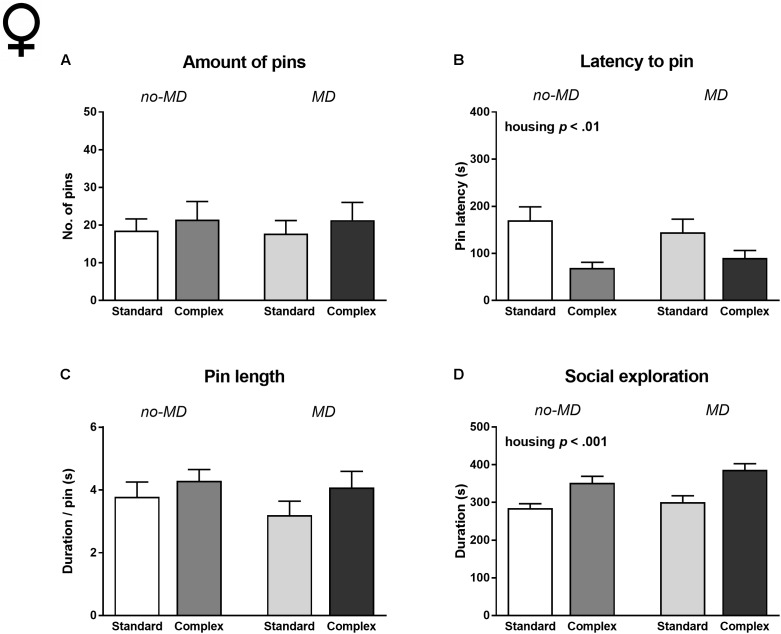
Female adolescent social play behavior following 24 h of social isolation in MD and complex housed rats. **(A)** Total amount of pins, **(B)** latency to pin, **(C)** pin length, and **(D)** time spent in social exploration. Statistics depict main effects. Graphs represent mean + SEM. Group sizes: no-MD standard housing *n* = 13, no-MD complex housing *n* = 13, MD standard housing *n* = 13, MD complex housing *n* = 12 couples.

All females showed social play behavior after 24 h of social isolation, but there was no effect of MD on any of the behavioral measures (**Figure 3** and **Supplementary Table S2**). In contrast to the males, the amount of pins and pin length was also unaffected by housing condition (**Figures 3A,C**). However, like males, complex housed females showed a shorter latency to engage in social play [*F*(1,47) = 11.96, *p* < 0.001, ${\text{η}}_{\text{p}}^{2}$ = 0.20; **Figure 3B**] and increased social exploration compared to standard housed females [*F*(1,47) = 23.14, *p* < 0.001, ${\text{η}}_{\text{p}}^{2}$ = 0.33; **Figure 3D**]. MD did not interact with the effects of housing.

### Adult Three-Chamber Social Approach Task

#### Habituation

During the habituation phase, none of the groups in both male and female experiments had a preference for either of the rooms. The mean DI per experimental group ranged from 44.9 to 50% in males and 45.6 to 51.5% in females [*t*(11–15) = -1.66 to 0.46, *p* = 0.13–1.0, range for all experimental groups tested against 50%]. Moreover, there were no effects of MD or complex housing on DI during habituation and no difference in approach latency between the two empty cylinders in any of the groups (see **Supplementary Tables S3**, **S4** for males and females, respectively).

#### Social Interest and Social Discrimination

Earlier experiments (van der Veen et al., 2015) showed that complex housed animals quickly start exploring a novel environment and habituate faster than standard housed animals. During the social interest phase, we analyzed the 5-min data over time and indeed saw high levels of initial activity and a more rapid decline in activity over the course of testing in complex compared to standard housed males, but not females [males: time ^∗^ housing *F*(1,60) = 11.69, *p* < 0.005, ${\text{η}}_{\text{p}}^{2}$ = 0.16, females: time ^∗^ housing *F*(1,55) = 0.01, *p* = 0.91, ${\text{η}}_{\text{p}}^{2}$ = 0.00, data not shown]. To avoid a habituation effect of complex housing on preference, we analyzed behavior in time bins. For graphical purposes we chose to plot the data in time bins of 2.5 min. Analysis of the data per minute yielded comparable results (data not shown).

#### Males

**FIGURE 4:**
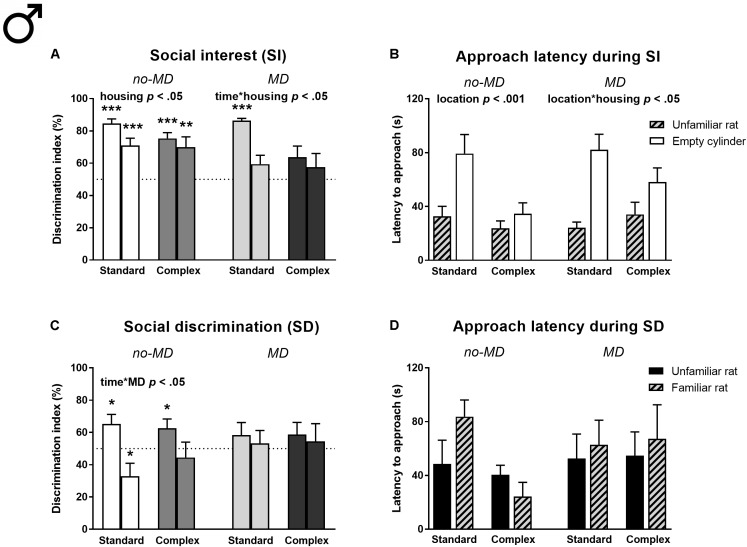
Male adult three-chamber social approach task in MD and complex housed rats. Social interest is represented by **(A)** the percentage of cylinder exploration time spent in the cylinder zone containing the unfamiliar rat and **(B)** the latency to approach both cylinders. Social discrimination is represented by **(C)** the percentage of cylinder exploration time spent in the cylinder zone containing the new unfamiliar rat and **(D)** the latency to approach both cylinders. Since activity declined over time, the 5-min task is split in two time bins in graphs **A,C**. Graphs represent mean + SEM. *n* = 16 males per experimental group. ^∗^*p* < 0.05, ^∗∗^*p* < 0.01, and ^∗∗∗^*p* < 0.001 indicate that DI is significantly different from chance (tested with a one-sample Student’s *t*-test against 50%).

Social interest (**Figures 4A,B** and **Supplementary Tables S3, S5**). In the social interest phase, complex housed rats spent less time with the unfamiliar rat [Housing *F*(1,60) = 8.01, *p* < 0.01, ${\text{η}}_{\text{p}}^{2}$ = 0.12, **Supplementary Table S3**] and showed a reduced preference for the cylinder zone containing an unfamiliar rat compared to standard housed males [*F*(1,60) = 4.79, *p* < 0.05, ${\text{η}}_{\text{p}}^{2}$ = 0.07], which was most pronounced during the first half of the test [DI time ^∗^ housing *F*(1,60) = 5.61, *p* < 0.05, ${\text{η}}_{\text{p}}^{2}$ = 0.09; **Figure 4A**]. MD did not significantly affect behavior. However, maternally deprived complex housed animals did not show a significant preference for either cylinder, not even in the first time bin (for results on all *t*-tests against 50% see **Supplementary Table S5**), unlike the other groups that showed a strong preference for the unfamiliar rat over the empty cylinder in the first half of the test.

All animals showed a shorter latency to approach the cylinder containing the stimulus rat compared to the empty cylinder [location *F*(1,60) = 25.41, *p* < 0.001, ${\text{η}}_{\text{p}}^{2}$ = 0.30; **Figure 4B**]. This difference was more pronounced in standard compared to complex housed animals [location ^∗^ housing *F*(1,60) = 6.31, *p* < 0.05, ${\text{η}}_{\text{p}}^{2}$ = 0.10]. The complex housing effects on social interest were observed in all groups, regardless of MD.

In this phase, all males spent time near the stimulus rat and had enough opportunity to get acquainted to this rat (no-MD standard housing 259.4 ± 14.8 s, no-MD complex housing 221.8 ± 20.9 s, MD standard housing 242.3 ± 14.5 s, MD complex housing 164.1 ± 17.0 s), which was a requirement for the social discrimination phase. However, the amount of time spent near the rat in the social interest phase did not correlate to the DI of the social discrimination phase (Pearson’s *r* = -0.09, *p* = 0.47).

Social discrimination (**Figures 4C,D** and **Supplementary Tables S3**, **S5**). In the social discrimination phase, MD influenced the DI over time [time ^∗^ MD *F*(1,60) = 4.37, *p* < 0.05, ${\text{η}}_{\text{p}}^{2}$ = 0.07; **Figure 4C**]: while no-MD animals showed a higher preference for the new unfamiliar rat in the first compared to the second time bin, this was not observed in the MD groups. Specifically, the no-MD groups showed a significant preference for the new unfamiliar rat in the first time bin, while they showed a preference for the familiar rat or no preference in the second time bin (see **Supplementary Table S5**). The maternally deprived animals did not show a preference at all in either of the time bins. Complex housing did not affect social discrimination, nor did it moderate the effect of MD. There were also no significant differences in approach latency between the cylinders containing a familiar or unfamiliar rat and neither MD nor housing influenced latency (**Figure 4D**).

#### Females

**FIGURE 5:**
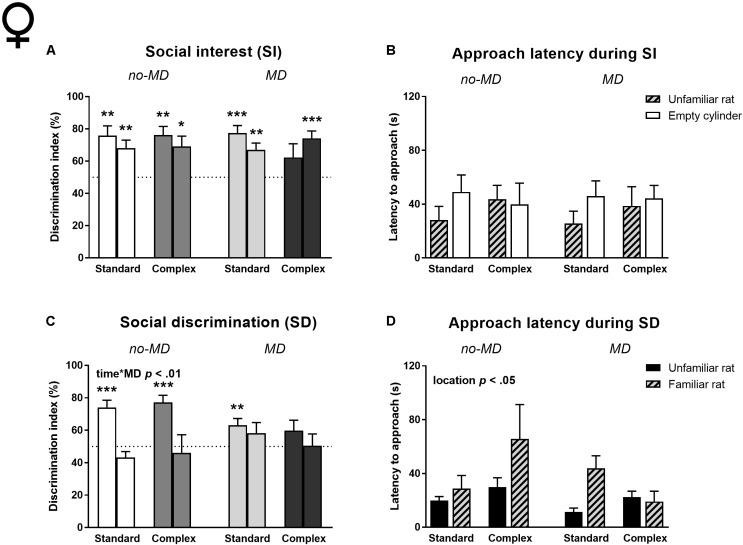
Female adult three-chamber social approach task in MD and complex housed rats. Social interest is represented by **(A)** the percentage of cylinder exploration time spent in the cylinder zone containing the unfamiliar rat and **(B)** the latency to approach both cylinders. Social discrimination is represented by **(C)** the percentage of cylinder exploration time spent in the cylinder zone containing the new unfamiliar rat and **(D)** the latency to approach both cylinders. Since activity declined over time, the 5-min task is split in two time bins. Graphs represent mean + SEM. Group sizes: no-MD standard housing *n* = 16, no-MD complex housing *n* = 12, MD standard housing *n* = 16, MD complex housing *n* = 15. ^∗^*p* < 0.05, ^∗∗^*p* < 0.01, and ^∗∗∗^*p* < 0.001 indicate that DI is significantly different from chance (tested with a one-sample Student’s *t*-test against 50%).

Social interest (**Figures 5A,B** and **Supplementary Tables S4**, **S5**). Neither MD, nor complex housing influenced the amount of time spent near the rat (**Supplementary Table S4**) or cylinder preference in the first 5 min (**Figure 5A** and **Supplementary Table S4**). Like the males, maternally deprived complex housed females did not show a preference for the unfamiliar rat in the first time bin, but unlike the males they did show this preference in the second time bin (for results on all *t*-tests against 50% see **Supplementary Table S5**). All other female groups preferred the unfamiliar rat over the empty cylinder in both time bins. No differences in approach latency between the empty and unfamiliar rat containing cylinders were observed and approach latencies were not influenced by MD or housing condition (**Figure 5B** and **Supplementary Table S4**).

All females spent time near the stimulus rat during the social interest phase (no-MD standard housing 242.6 ± 11.2 s, no-MD complex housing 258.5 ± 23.5 s, MD standard housing 259.2 ± 12.8 s, MD complex housing 259.2 ± 15.2 s). The amount of time spent near the rat in the social interest phase did not correlate to the DI of the social discrimination phase (Pearson’s *r* = -0.12, *p* = 0.36).

Social discrimination (**Figures 5C,D** and **Supplementary Tables S4**, **S5**). Similar to the male results, MD influenced the DI over time [time ^∗^ MD *F*(1,55) = 7.27, *p* < 0.01, ${\text{η}}_{\text{p}}^{2}$ = 0.12; **Figure 5C**]; no-MD animals showed a higher preference for the new, unfamiliar rat in the first compared to the second time bin, which was not observed in the MD groups. The maternally deprived complex housed females did not show a preference in either of the time bins. All other groups showed a preference for the unfamiliar rat in the first time bin (**Supplementary Table S5**). Complex housing did not influence social discrimination, nor did it moderate MD effects. There was a significant difference in approach latency between the cylinders, with an overall shorter latency to approach the cylinder containing the unfamiliar rat compared to the familiar rat [location *F*(1,55) = 5.94, *p* < 0.05, ${\text{η}}_{\text{p}}^{2}$ = 0.10; **Figure 5D**]. This was predominantly seen in the maternally deprived standard housed animals [location ^∗^ MD ^∗^ housing *F*(1,55) = 4.03, *p* = 0.05, ${\text{η}}_{\text{p}}^{2}$ = 0.07].

## Discussion

In a design with MD (vs. no deprivation) on PND 3 and complex (vs. standard) housing starting at PND 26, we found that adolescent maternally deprived males, but not females, showed a longer latency to engage in social play behavior and a decreased total amount of social play behavior after a 24 h isolation period compared to non-deprived animals (see **Table 3** for a schematic overview of all results). In adulthood, social interest was not affected, but both male and female maternally deprived rats showed impaired social discrimination. Complex housing independently affected social behavior. Adolescent complex housed males and females showed very little social play behavior after 3 h of social isolation, suggesting a lack of motivation to engage in social play behavior due to the complex housing conditions. However, after a 24 h isolation period, complex housed males showed more social play behavior, while both males and females showed a shorter latency to play and increased social exploration compared to standard housed animals. In adulthood, male complex housed animals showed a decreased social interest at the start of testing. Complex housing did not moderate the effects of MD in adolescence or adulthood.

**Table 3:** Schematic overview of main effects of maternal deprivation and complex housing on adolescent and adult body weight and social behavior.

Variables		Maternal deprivation		Complex housing	
		Males	Females	Males	Females
Adolescence	Body weight	↓	↓	↓	=
	Social play after 3 h isolation	=	=	↓	↓
	Social play after 24 h isolation	↓	=	↑	=
Adulthood	Body weight	=	↓	=	↓
	Social interest	=	=	↓	=
	Social discrimination	↓	↓	=	=

*“↑” improved/higher, “↓” impaired/lower or “ = ” no change compared to control conditions (MD vs. no-MD; complex vs. standard housing)*.

Consistent with previous studies (Marco et al., 2015; van der Veen et al., 2015; Kentrop et al., 2016), maternally deprived animals showed a lower body weight in adolescence. While deprived males were able to catch up with non-deprived males in adulthood, the weight difference persisted in females. During MD, pups are deprived of both maternal care and nutrition for 24 h. With the current model these two factors cannot be separated and it is possible that both factors contribute to the diminished body weight gain. Independent of early life conditions, complex housing also lowered body weight in both males and females [as we and others have seen before (Moncek et al., 2004; Peña et al., 2006; Harati et al., 2013; van der Veen et al., 2015)]. However, males responded to the change in housing with a lowered body weight directly after the start of complex housing but not adulthood, while complex housed females revealed the reduction in body weight only in adulthood.

In adolescence, MD effects on social play behavior were found in males, but not females. After 24 h of social isolation, deprived males showed longer latencies to play and less social play bouts compared to no-MD males. In contrast, social exploration was higher in deprived males. This might indicate that MD specifically reduces social play behavior, but not general social interest. In the literature both increasing and decreasing effects of ELS on play behavior have been reported (after various isolation lengths before testing) (Arnold and Siviy, 2002; Veenema and Neumann, 2009; Muhammad and Kolb, 2011; Zimmerberg and Sageser, 2011; Van Hasselt et al., 2012; Lundberg et al., 2017). Similar to our study, effects were mainly found in males, while females seem to be more resilient (but see Zimmerberg and Sageser, 2011).

Compared to standard housed males, complex housed males showed more social play behavior and an increased pin length. In a study conducted by Panksepp (1981), rats that were allowed to play with the same play partner multiple times over a course of 40 days, developed a stable hierarchy over time in which the more dominant partner exhibited longer pins compared to the submissive partner. However, in our study we recorded pin length per couple (i.e., no individual data) and play partners were unfamiliar. Nonetheless, complex housed animals are reared in an environment in which the social hierarchy is undoubtedly more complex than the dominance relationship between pairs of standard housed rats and it is not unlikely that this is reflected in pin length.

Both complex housed males and females needed less time to engage in social play after 24 h of isolation. Previously we and others have shown that complex housed rats habituate faster to novel situations (Zimmermann et al., 2001; Moncek et al., 2004; van der Veen et al., 2015), which might explain why they engage in social play sooner with an unfamiliar play partner. Besides an enhancement of social play behavior in males, both male and female complex housed rats also showed increased social exploration of their play partners, suggesting that complex housing – different from deprivation – has a positive influence on both play- and non-play-related social behaviors.

The effect of complex housing on social play behavior strongly depended on the length of social isolation before the test. Commonly, rats are socially isolated for 3–24 h before social play testing to increase their play motivation. During isolation, rats are deprived of a play partner and hence play more when the opportunity presents itself. Indeed, up to 24 h, longer periods of isolation induce more play (Panksepp and Beatty, 1980), but see Manduca et al. (2016) for an exception. Isolation can be viewed as stressful, since rats are deprived of their cage mate and exposed to a novel environment. In that view, playing after social isolation could be seen as a relief from stress for the rats. To our surprise 3 h of social isolation did not induce social play behavior at all in our complex housed rats, while they displayed abundant amounts of social play after 24 h isolation. Complex housed animals may not have been affected by 3 h social isolation as much as standard housed animals, and consequently did not show as much social play. This is supported by the observation that rats living in complex housing not only habituate faster to novelty (Zimmermann et al., 2001; Moncek et al., 2004; van der Veen et al., 2015), but also show a decreased HPA-axis response to a variety of stressors (reviewed in Simpson and Kelly, 2011).

In adulthood, social interest was not affected by MD. This is in line with the results obtained on social exploration in adolescence, showing an absence of MD effects under standard housing conditions. Others have reported that social interest in adult rats was either not affected (Hulshof et al., 2011; Zamberletti et al., 2012; Zhang et al., 2014) or reduced (Maciag et al., 2002; Takase et al., 2012) by ELS. Such divergence of effects may not be simply explained by differences in day of deprivation or the way of testing social interest, since a 24 h MD on PND 9 equally did not affect social interaction in an open arena where both test and stimulus rats were freely moving and able to physically interact (Zamberletti et al., 2012). However, in this freely moving set-up, deprived animals were found to show increased aggression toward the unfamiliar stimulus rat (Zamberletti et al., 2012). In our three-chamber set-up, stimulus rats are positioned in a cylinder with bars, which prevents physical forms of aggression. It also assures that the amount of social interaction is fully driven by the initiative of the test subject and hence allows for a more precise measurement of preference (Camats Perna and Engelmann, 2017). Takase et al. (2012) tested male and female rats that underwent 12 h maternal separation on PND 9 and 11 in both types of social interest tests, with and without separation of stimulus rats, and found in both tests a reduction of social interest in males, but not females.

We found clear effects of MD on social discrimination in both sexes. In contrast to non-deprived animals, deprived animals did not discriminate between the unfamiliar and familiar rat. Social discrimination relies on the ability to recognize previously encountered conspecifics and contains a (short-term) memory component. The observed failure of MD rats to discriminate is not likely to be explained by a lack of familiarization in the social interest phase because the amount of time spent with the stimulus rat during this phase was not correlated with the DI in the social discrimination phase. This suggests that MD does not affect general social interest but impairs memory-related social behavior. ELS – using a variety of models (Krugers and Joëls, 2014; Tractenberg et al., 2016) – is known to impair (non-stressful) learning and memory. Our data are in line with these results and support that MD-induced impairments in learning and memory also include learning and memory of social information.

Complex housed males showed the opposite picture, that is, less social interest but a comparable social discrimination as standard housed animals; adult female social behavior was not affected by complex housing. A diminished social interest in males is in line with the lack of social play behavior after 3 h of isolation in adolescence, but contrasts with the increased amount of social play after 24 h of isolation. As with adolescent social play, there were no interaction effects with MD in adult social behavior. So, while complex housing treatment induced a distinct behavioral phenotype in adolescence and adulthood, it did not rescue MD-induced impairments in social behavior. Environmental enrichment has been shown to successfully reverse several ELS-induced impairments in behavior, including learning and memory, anxiety, depressive-like behavior, and attention (Francis et al., 2002; Cui et al., 2006; Vivinetto et al., 2013; Zubedat et al., 2015; Koe et al., 2016). Notably, Morley-Fletcher et al. (2003) were able to diminish prenatal stress-induced impairments in social play behavior in rats with physical, but not social, environmental enrichment (Laviola et al., 2004). Complex housing has three key components that each contribute to the phenotype; social enrichment, physical enrichment, and repeated exposure to novelty. These aspects can be applied in several combinations and varied intensities and likely each has a different impact, possibly across different behavioral domains. Brenes et al. (2016), for example, found that social enrichment increased social approach behavior in response to the play-back of prosocial 50 kHz calls in a radial maze, while physical enrichment led to a reduction in social approach. This would be partly in line with our results. Of note, social enrichment without physical enrichment might turn into crowding if the available space per rat becomes too constricted. In order to correctly interpret our current findings and compare them across studies, not only differences in the ELS model, but also different key components of enrichment should be systematically varied and studied as separate factors.

Despite the observation that MD and complex housing seem to affect both adolescent social play behavior and adult social behavior independently (no interaction effects), we cannot ignore that it is the complex housed maternally deprived group that mainly shows deprivation effects in adolescent males (amount of social play, social exploration) and that this is also the only group that does not show social interest in adult males or social discrimination in adult females. The current data set seems to lack sufficient power to statistically support this qualitative observation. Validation of this finding might be accomplished by performing a meta-analysis on this and comparable studies and/or a replication of the current experiments with larger experimental groups.

Males seem more affected by the environmental changes in this study compared to females. Males and females seem to be differently susceptible to ELS-associated pathologies, but it depends on the timing of stressors (prenatal, postnatal, adolescent) whether males or females are more affected [see Bale and Epperson (2015) for a very elegant overview]. Focusing on early postnatal manipulations of maternal care in rodents as a model for ELS, it seems that males are more affected than females in a broad range of behavioral domains (Loi et al., 2017; Schroeder et al., 2018). One potential explanation is that males, compared to females, receive more maternal care from their mothers (Moore and Morelli, 1979; Van Hasselt et al., 2012). As a consequence, any model depriving pups of maternal care might be more severe for males than females. We could not compare males and females in the same analysis, however, since experiments were performed separately.

A prominent feature in the phenotype of our complex housed rats, and a recurrent theme throughout this paper, is the behavioral response to novel situations. Whenever rodents encounter new situations, there is a trade-off between the natural tendency to explore and an innate anxiety for novelty. Both in adolescence and adulthood, complex housed rats showed shorter latencies to approach novel conspecifics and objects, which suggests a decrease in novelty anxiety. Crofton et al. (2015) use the inoculation stress hypothesis to explain the phenotypic changes typically seen in complex housed animals. They propose that living in a complex environment places animals in a state of chronic very mild stress that inoculates against subsequent stressors. If true, this might explain why complex housed rats seem more resilient to the arousal that is caused by novel situations, which might lead to unexpected behavioral results, like the lack of play after a 3 h isolation period. We therefore recommend extra caution with the interpretation of novelty containing tests conducted outside of the home cage, regardless of the type of behavior that is under investigation.

Taken together, this study provides support for three main conclusions. First, MD has a negative impact on social behavior in both adolescent and adult rats, but the effects are modest. It remains to be determined if this applies to the specific type of ELS model applied here, or if social behavior compared to behavior in, e.g., the cognitive or anxiety domains is less vulnerable to early life stressors in this particular time window of development. Second, complex housing induces a strong behavioral phenotype, with a lack of social play behavior after 3 h social isolation or showing shorter latencies to engage in social play behavior and increased pin length when longer isolation periods are used. In adulthood these animals showed less social interest, but intact social discrimination. In general, complex housed animals appear to quickly integrate a novel environment (van der Veen et al., 2015) and, probably related to this, seem to be differentially influenced by novel testing conditions. This might explain the low interest in playing after a short isolation period, while vigorously playing after having spent 24 h alone. Finally, based on the current data, we conclude that complex housing is not a suitable intervention to reverse or diminish the effects of early life adversity on social behavior. MD and complex housing seem to exert their effects on behavior through different mechanisms that do not seem to interact. However, as complex housing affected several behavioral measures indicative of a fast integration of the environment, this phenotype is likely advantageous in other behavioral domains such as cognition and anxiety. These domains are well known to be affected by ELS and complex housing might be a promising intervention strategy for ELS-induced impairments in cognition and/or anxiety-related behaviors.

## Author Contributions

Authors have made substantial contributions to the following: JK, EA, MvI, MB-K, MJ, and RvdV conception and design of the study, interpretation of data, and final approval of the version to be submitted. JK and CS acquisition of data. JK, MvI, MB-K, MJ, and RvdV analysis of data and drafting the article critically for important intellectual content. JK, CS, EA, MvI, MB-K, MJ, and RvdV agreement to be accountable for all aspects of the work ensuring that questions related to the accuracy or integrity of any part of the work are appropriately investigated and resolved.

## Conflict of Interest Statement

The authors declare that the research was conducted in the absence of any commercial or financial relationships that could be construed as a potential conflict of interest.
