# Supplementary material for: Effects of Maternal Deprivation and Complex Housing on Rat Social Behavior in Adolescence and Adulthood
Source: Front Behav Neurosci. 2018 Sep 11;12:193. doi: 10.3389/fnbeh.2018.00193 (PMC6141926; doi:10.3389/fnbeh.2018.00193)
Supplement: Supplementary file 1 [file Data_Sheet_1.PDF]

Supplementary data

Supplementary table S1: Adolescent social play after 3h of social isolation in maternally deprived (MD) and complex housed male and female rats. Numbers represent mean ± SEM and *p* values in bold are considered statistically significant. Group sizes males: *n* = 8 couples for all groups, females: *n* = 8 no-MD standard housing, *n* = 7 no-MD complex housing, *n* = 7 MD standard housing, *n* = 8 MD complex housing.

Variables		No-MD		MD		Test	Effect	Degrees of freedom	*p*	η_p^2
		Standard housing	Complex housing	Standard housing	Complex housing					
**Males**	Amount of pins (#)	16.50 ± 4.11	3.00 ± 1.30	16.88 ± 3.75	2.38 ± 1.05	2x2 ANOVA	MD	F(1,28) = 0.00	.966	.00
							Housing	F(1,28) = 23.22	**<.001**	.45
							MD*Housing	F(1,28) = 0.03	.865	.00
	Latency to pin (s)	225.54 ± 97.56	569.75 ± 114.85	191.95 ± 101.98	578.63 ± 126.30	2x2 ANOVA	MD	F(1,28) = 0.01	.912	.00
							Housing	F(1,28) = 10.89	**.003**	.28
							MD*Housing	F(1,28) = 0.04	.849	.00
	Pin length (duration/pin in s)	4.65 ± 0.35	5.74 ± 0.95	3.67 ± 0.25	6.85 ± 1.69	2x2 ANOVA	MD	F(1,21) = 0.01	.935	.00
							Housing	F(1,21) = 6.75	**.017**	.24
							MD*Housing	F(1,21) = 1.61	.219	.07
	Social exploration (s)	227.20 ± 16.91	270.09 ± 28.00	267.89 ± 29.85	265.63 ± 31.26	2x2 ANOVA	MD	F(1,28) = 0.45	.509	.02
							Housing	F(1,28) = 0.56	.460	.02
							MD*Housing	F(1,28) = 0.69	.412	.02
	Social rest (s)	0.00 ± 0.00	33.43 ± 16.76	0.00 ± 0.00	90.16 ± 24.93	2x2 ANOVA	MD	F(1,28) = 3.57	.069	.11
							Housing	F(1,28) = 16.93	**<.001**	.38
							MD*Housing	F(1,28) = 3.57	.069	.11
**Females**	Amount of pins (#)	16.25 ± 3.50	0.00 ± 0.00	14.43 ± 4.73	0.00 ± 0.00	2x2 ANOVA	MD	F(1,26) = 0.10	.755	.00
							Housing	F(1,26) = 28.11	**<.001**	.52
							MD*Housing	F(1,26) = 0.10	.755	.00
	Latency to pin (s)	217.72 ± 99.12	n/a	190.39 ± 44.65	n/a	2x2 ANOVA	MD	F(1,26) = 0.06	.813	.00
							Housing	F(1,26) = 148.33	**<.001**	.85
							MD*Housing	F(1,26) = 0.06	.813	.00
	Pin length (duration/pin in s)	3.36 ± 0.36	n/a	4.01 ± 0.55	n/a	2x2 ANOVA	MD	F(1,12) = 0.996	.338	.08
							Housing	n/a	n/a	n/a
							MD*Housing	n/a	n/a	n/a
	Social exploration (s)	253.59 ± 18.94	312.48 ± 50.32	276.01 ± 11.70	297.58 ± 20.64	2x2 ANOVA	MD	F(1,26) = 0.02	.896	.00
							Housing	F(1,26) = 2.01	.168	.07
							MD*Housing	F(1,26) = 0.43	.516	.02
	Social rest (s)	0.00 ± 0.00	30.32 ± 19.42	0.00 ± 0.00	85.89 ± 33.29	2x2 ANOVA	MD	F(1,26) = 1.92	.177	.07
							Housing	F(1,26) = 8.41	**.007**	.24
							MD*Housing	F(1,26) = 1.92	.177	.07

Supplementary table S2: Adolescent social play after 24h of social isolation in maternally deprived (MD) and complex housed male and female rats. Numbers represent mean ± SEM and *p* values in bold are considered statistically significant. No social rest was observed during play after 24h isolation. Group sizes males *n* = 10 couples for all groups, females: *n* = 13 no-MD standard housing, *n* = 13 no-MD complex housing, *n* = 13 MD standard housing, *n* = 12 MD complex housing.

**Variables**		**No-MD**		**MD**		**Test**	**Effect**	**Degrees of freedom**	***p***	η_p^2
		**Standard housing**	**Complex housing**	**Standard housing**	**Complex housing**					
**Males**	Amount of pins (#)	19.60 ± 3.96	38.20 ± 4.82	16.70 ± 3.70	22.50 ± 3.23	2x2 ANOVA	MD	F(1,36) = 5.49	**.025**	.13
							Housing	F(1,36) = 9.44	**.004**	.21
							MD*Housing	F(1,36) = 2.60	.116	.07
	Latency to pin (s)	144.29 ± 24.05	43.94 ± 7.51	223.04 ± 47.53	111.77 ± 30.17	2x2 ANOVA	MD	F(1,36) = 5.65	**.023**	.14
							Housing	F(1,36) = 11.77	**.002**	.25
							MD*Housing	F(1,36) = 0.03	.860	.00
	Pin length (duration/pin in s)	1.87 ± 0.16	2.98 ± 0.28	2.04 ± 0.11	2.78 ± 0.22	2x2 ANOVA	MD	F(1,36) = 0.00	.949	.00
							Housing	F(1,36) = 20.67	**<.001**	.37
							MD*Housing	F(1,36) = 0.88	.354	.02
	Social exploration (s)	260.21 ± 12.43	284.23 ± 13.21	272.41 ± 13.12	351.60 ± 27.05	2x2 ANOVA	MD	F(1,36) = 5.13	**.030**	.13
							Housing	F(1,36) = 8.64	**.006**	.19
							MD*Housing	F(1,36) = 2.47	.125	.06
**Females**	Amount of pins (#)	18.54 ± 3.11	21.46 ± 4.80	17.77 ± 3.44	21.33 ± 4.68	2x2 ANOVA	MD	F(1,47) = 0.01	.912	.00
							Housing	F(1,47) = 0.64	.428	.01
							MD*Housing	F(1,47) = 0.06	.937	.00
	Latency to pin (s)	170.25 ± 28.67	68.92 ± 12.28	145.03 ± 27.64	90.55 ± 15.78	2x2 ANOVA	MD	F(1,47) = 0.01	.937	.00
							Housing	F(1,47) = 11.96	**.001**	.20
							MD*Housing	F(1,47) = 1.08	.304	.02
	Pin length (duration/pin in s)	3.78 ± 0.47	4.29 ± 0.36	3.20 ± 0.44	4.07 ± 0.52	2x2 ANOVA	MD	F(1,47) = 0.79	.379	.02
							Housing	F(1,47) = 2.34	.131	.05
							MD*Housing	F(1,47) = 0.16	.688	.00
	Social exploration (s)	284.74 ± 11.73	352.07 ± 17.04	300.53 ± 17.33	386.07 ± 16.85	2x2 ANOVA	MD	F(1,47) = 2.45	.124	.05
							Housing	F(1,47) = 23.14	**<.001**	.33
							MD*Housing	F(1,47) = 0.33	.569	.01

Supplementary table S3: Male adult social interaction measured with the three-chamber social approach task. Numbers represent mean ± SEM and *p* values in bold are considered statistically significant. Group sizes *n* = 16 animals in all groups.

**Variables**	**No-MD**		**MD**		**Test**	**Effect**	**Degrees of freedom**	***p***	η_p^2
	Standard housing	Complex housing	Standard housing	Complex housing					
Habituation: Discrimination index rooms (%)	47.39 ± 4.41	48.42 ± 2.26	48.61 ± 3.78	44.87 ± 3.60	2x2 ANOVA	MD	F(1,60) = 0.82	.369	.01
						Housing	F(1,60) = 0.11	.743	.00
						MD*Housing	F(1,60) = 0.35	.556	.01
Habituation: Approach latency empty cylinders (s)	Cylinder 1: 46.30 ± 9.27 Cylinder 2: 39.81 ± 10.41	Cylinder 1: 27.49 ± 5.30 Cylinder 2: 17.76 ± 6.21	Cylinder 1: 17.32 ± 4.08 Cylinder 2: 32.47 ± 7.05	Cylinder 1: 33.67 ± 8.88 Cylinder 2: 25.17 ± 6.29	2x2 Repeated measures ANOVA	MD	F(1,60) = 2.31	.134	.04
						Housing	F(1,60) = 4.53	**.037**	.07
						MD*Housing	F(1,60) = 11.14	**.001**	.16
						Location	F(1,60) = 0.14	.712	.00
						Location*MD	F(1,60) = 0.78	.380	.01
						Location*Housing	F(1,60) = 1.08	.302	.02
						Location*MD*Housing	F(1,60) = 0.62	.433	.01
Social interest: Time spent with rat (s) (0-5 min)	141.43 ± 8.18	123.79 ± 7.70	132.32 ± 7.50	96.73 ± 13.09	2x2 ANOVA	MD	F(1,60) = 3.70	.059	.06
						Housing	F(1,60) = 8.01	**.006**	.12
						MD*Housing	F(1,60) = 0.91	.344	.02
Social interest: Discrimination index (%) (0-5 min)	0-2.5 min: 84.55 ± 2.83 2.5-5 min: 70.93 ± 4.54	0-2.5 min: 75.34 ± 3.60 2.5-5 min: 69.92 ± 6.43	0-2.5 min: 86.33 ± 1.41 2.5-5 min: 59.38 ± 5.51	0-2.5 min: 63.74 ± 6.92 2.5-5 min: 57.51 ± 8.49	2x2 Repeated measures ANOVA	MD	F(1,60) = 3.69	.059	.06
						Housing	F(1,60) = 4.79	**.033**	.07
						MD*Housing	F(1,60) = 0.94	.335	.02
						Time	F(1,60) = 18.29	**<.001**	.23
						Time*MD	F(1,60) = 1.34	.251	.02
						Time*Housing	F(1,60) = 5.61	**.021**	.09
						Time*MD*Housing	F(1,60) = 1.05	.309	.02
Social interest: Approach latency (s)	Rat: 32.77 ± 7.30 Empty: 79.32 ± 14.11	Rat: 23.79 ± 5.51 Empty: 34.67 ± 8.07	Rat: 24.14 ± 4.30 Empty: 82.13 ± 11.52	Rat: 34.09 ± 9.09 Empty: 58.22 ± 10.45	2x2 Repeated measures ANOVA	MD	F(1,60) = 1.28	.263	.02
						Housing	F(1,60) = 7.42	**.008**	.11
						MD*Housing	F(1,60) = 2.56	.115	.04
						Location	F(1,60) = 25.41	**<.001**	.30
						Location*MD	F(1,60) = 0.80	.376	.01
						Location*Housing	F(1,60) = 6.31	**.015**	.10
						Location*MD*Housing	F(1,60) = 0.00	.948	.00
Social discrimination: Discrimination index (%)	0-2.5 min: 65.28 ± 5.92 2.5-5 min: 32.87 ± 8.03	0-2.5 min: 62.58 ± 5.76 2.5-5 min: 44.50 ± 9.53	0-2.5 min: 58.38 ± 7.81 2.5-5 min: 53.14 ± 8.05	0-2.5 min: 58.71 ± 7.54 2.5-5 min: 54.48 ± 10.95	2x2 Repeated measures ANOVA	MD	F(1,60) = 0.63	.432	.01
						Housing	F(1,60) = 0.29	.594	.01
						MD*Housing	F(1,60) = 0.00	.995	.00
						Time	F(1,60) = 9.30	**.003**	.13
						Time*MD	F(1,60) = 4.37	**.041**	.07
						Time*Housing	F(1,60) = 0.61	.439	.01
						Time*MD*Housing	F(1,60) = 0.46	.500	.01
Social discrimination: Approach latency (s)	Unfamiliar: 41.08 ± 10.72 Familiar: 83.69 ± 12.42	Unfamiliar: 40.49 ± 7.05 Familiar: 24.36 ± 10.49	Unfamiliar: 45.48 ± 12.53 Familiar: 62.39 ± 18.08	Unfamiliar: 47.14 ± 11.23 Familiar: 66.59 ± 24.96	2x2 Repeated measures ANOVA	MD	F(1,60) = 0.74	.393	.01
						Housing	F(1,60) = 2.11	.151	.03
						MD*Housing	F(1,60) = 3.13	.082	.05
						Location	F(1,60) = 2.03	.159	.03
						Location*MD	F(1,60) = 0.05	.824	.00
						Location*Housing	F(1,60) = 1.63	.207	.03
						Location*MD*Housing	F(1,60) = 1.93	.169	.03
Distance moved(cm): Habituation	2593 ± 149	3024 ± 133	2711 ± 128	2917 ± 140	2x2 Repeated measures ANOVA	MD	F(1,60) = 2.28	.137	.04
						Housing	F(1,60) = 0.83	.366	.01
						MD*Housing	F(1,60) = 1.73	.194	.03
Distance moved(cm): Social interest	2452 ± 121	2530 ± 102	2439 ± 90	2141 ± 94		Time	F(2,120) = 232.88	**<.001**	.80
						Time*MD	F(2,120) = 1.64	.198	.03
Distance moved(cm): Social discrimination	1740 ± 117	1336 ± 68	1585 ± 104	1099 ± 103		Time*Housing	F(2,120) = 17.29	**<.001**	.22
						Time*MD*Housing	F(2,120) = 0.64	.531	.01

Supplementary table S4: Female adult social interaction measured with the three-chamber social approach task. Numbers represent mean ± SEM and *p* values in bold are considered statistically significant. Group sizes: *n* = 16 animals no-MD standard housing, *n* = 12 no-MD complex housing, *n* = 16 MD standard housing, *n* = 15 MD complex housing.

**Variables**	**No-MD** Standard housing	**No-MD** Complex housing	**MD** Standard housing	**MD** Complex housing	**Test**	**Effect**	**Degrees of freedom**	***p***	η_p^2
Habituation: Discrimination index rooms (%)	47.43 ± 3.29	45.61 ± 3.80	49.42 ± 3.29	51.53 ± 3.40	2x2 ANOVA	MD	F(1,55) = 1.32	.256	.02
						Housing	F(1,55) = 0.00	.967	.00
						MD*Housing	F(1,55) = 0.33	.570	.01
Habituation: Approach latency empty cylinders (s)	Cylinder 1: 28.16 ± 5.67 Cylinder 2: 24.16 ± 6.68	Cylinder 1: 13.55 ± 4.54 Cylinder 2: 17.63 ± 3.88	Cylinder 1: 17.53 ± 4.98 Cylinder 2: 28.98 ± 6.81	Cylinder 1: 16.85 ± 4.59 Cylinder 2: 13.85 ± 3.90	2x2 Repeated measures ANOVA	MD	F(1,55) = 0.35	.557	.01
						Housing	F(1,55) = 12.03	**.001**	.18
						MD*Housing	F(1,55) = 0.25	.619	.01
						Location	F(1,55) = 0.20	.657	.00
						Location*MD	F(1,55) = 0.19	.663	.00
						Location*Housing	F(1,55) = 0.11	.740	.00
						Location*MD*Housing	F(1,55) = 1.39	.243	.03
Social interest: Time spent with rat (s) (0-5 min)	142.99 ± 8.14	140.14 ± 9.63	147.02 ± 7.15	145.77 ± 10.37	2x2 ANOVA	MD	F(1,55) = 0.30	.588	.01
						Housing	F(1,55) = 0.05	.818	.00
						MD*Housing	F(1,55) = 0.01	.929	.00
Social interest: Discrimination index (%) (0-5 min)	0-2.5 min: 75.82 ± 6.01 2.5-5 min: 68.03 ± 4.98	0-2.5 min: 76.09 ± 5.38 2.5-5 min: 69.14 ± 6.30	0-2.5 min: 77.39 ± 4.68 2.5-5 min: 66.98 ± 4.16	0-2.5 min: 62.29 ± 8.46 2.5-5 min: 74.11 ± 4.57	2x2 Repeated measures ANOVA	MD	F(1,55) = 0.30	.584	.01
						Housing	F(1,55) = 0.46	.500	.01
						MD*Housing	F(1,55) = 0.02	.890	.00
						Time	F(1,55) = 0.58	.450	.01
						Time*MD	F(1,55) = 0.85	.360	.02
						Time*Housing	F(1,55) = 1.74	.193	.03
						Time*MD*Housing	F(1,55) = 1.50	.227	.03
Social interest: Approach latency (s)	Rat: 28.22 ± 10.10 Empty: 49.09 ± 12.62	Rat: 43.72 ± 10.25 Empty: 39.83 ± 15.74	Rat: 25.71 ± 9.08 Empty: 46.00 ± 11.26	Rat: 38.68 ± 14.31 Empty: 44.31 ± 9.57	2x2 Repeated measures ANOVA	MD	F(1,55) = 0.06	.802	.00
						Housing	F(1,55) = 0.52	.476	.01
						MD*Housing	F(1,55) = 0.04	.837	.00
						Location	F(1,55) = 1.14	.291	.02
						Location*MD	F(1,55) = 0.05	.825	.00
						Location*Housing	F(1,55) = 0.96	.332	.02
						Location*MD*Housing	F(1,55) = 0.06	.803	.00
Social discrimination: Discrimination index (%)	0-2.5 min: 74.05 ± 4.43 2.5-5 min: 43.13 ± 3.66	0-2.5 min: 77.08 ± 4.47 2.5-5 min: 46.08 ± 11.13	0-2.5 min: 63.15 ± 4.07 2.5-5 min: 58.09 ± 6.65	0-2.5 min: 59.72 ± 6.42 2.5-5 min: 50.34 ± 7.37	2x2 Repeated measures ANOVA	MD	F(1,55) = 1.85	.180	.03
						Housing	F(1,55) = 0.14	.706	.00
						MD*Housing	F(1,55) = 2.17	.146	.04
						Time	F(1,55) = 18.80	**<.001**	.26
						Time*MD	F(1,55) = 7.27	**.009**	.12
						Time*Housing	F(1,55) = 0.06	.804	.00
						Time*MD*Housing	F(1,55) = 0.06	.811	.00
Social discrimination: Approach latency (s)	Unfamiliar: 19.76 ± 3.10 Familiar: 28.71 ± 9.76	Unfamiliar: 29.62 ± 7.01 Familiar: 55.56 ± 18.10	Unfamiliar: 11.38 ± 2.92 Familiar: 43.82 ± 9.36	Unfamiliar: 22.41 ± 4.40 Familiar: 19.10 ± 7.66	2x2 Repeated measures ANOVA	MD	F(1,55) = 3.01	.088	.05
						Housing	F(1,55) = 1.17	.284	.02
						MD*Housing	F(1,55) = 5.60	**.021**	.09
						Location	F(1,55) = 5.94	**.018**	.10
						Location*MD	F(1,55) = 0.05	.827	.00
						Location*Housing	F(1,55) = 0.51	.479	.01
						Location*MD*Housing	F(1,55) = 4.03	.050	.07
Distance moved (cm): Habituation	3298 ± 179	3483 ± 118	3515 ± 128	3603 ± 126	2x2 Repeated measures ANOVA	MD	F(1,55) = 1.80	.185	.03
						Housing	F(1,55) = 15.52	**<.001**	.22
						MD*Housing	F(1,55) = 0.11	.743	.00
Distance moved (cm): Social interest	3275 ± 152	2944 ± 205	3353 ± 101	2961 ± 177		Time	F(2,110) = 61.09	**<.001**	.53
						Time*MD	F(2,110) = 0.60	.551	.01
Distance moved (cm): Social discrimination	2951 ± 98	1889 ± 191	3214 ± 162	2091 ± 122		Time*Housing	F(2,110) = 25.83	**<.001**	.32
						Time*MD*Housing	F(2,110) = 0.01	.992	.00

Supplementary table S5: Student's t-test against 50% chance level for discrimination index values in the three-chamber social approach task in male and female rats. Numbers represent mean ± SEM and *p* values in bold are considered statistically significant. Group sizes males: *n* = 16 animals in all groups and females: *n* = 16 no-MD standard housing, *n* = 12 no-MD complex housing, *n* = 16 MD standard housing, *n* = 15 MD complex housing.

<table>
<tr><th colspan="3" rowspan="2">Variables</th><th colspan="2">0-2.5min</th><th colspan="2">2.5-5min</th></tr>
<tr><th>Degrees of freedom</th><th>p</th><th>Degrees of freedom</th><th>p</th></tr>
<tr><td rowspan="8">Males</td><td rowspan="4">Social interest: Discrimination index (%)</td><td>No-MD Standard housing</td><td>t(15) = 12.22</td><td><.001</td><td>t(15) = 4.61</td><td><.001</td></tr>
<tr><td>No-MD Complex housing</td><td>t(15) = 7.04</td><td><.001</td><td>t(15) = 3.10</td><td>.007</td></tr>
<tr><td>MD Standard housing</td><td>t(15) = 25.79</td><td><.001</td><td>t(15) = 1.70</td><td>.109</td></tr>
<tr><td>MD Complex housing</td><td>t(15) = 1.99</td><td>.066</td><td>t(15) = 0.89</td><td>.390</td></tr>
<tr><td rowspan="4">Social discrimination: Discrimination index (%)</td><td>No-MD Standard housing</td><td>t(15) = 2.58</td><td>.021</td><td>t(15) = -2.13</td><td>.050</td></tr>
<tr><td>No-MD Complex housing</td><td>t(15) = 2.19</td><td>.045</td><td>t(15) = -.58</td><td>.573</td></tr>
<tr><td>MD Standard housing</td><td>t(15) = 1.07</td><td>.302</td><td>t(15) = 0.39</td><td>.702</td></tr>
<tr><td>MD Complex housing</td><td>t(15) = 1.16</td><td>.266</td><td>t(15) = 0.41</td><td>.688</td></tr>
<tr><td rowspan="8">Females</td><td rowspan="4">Social interest: Discrimination index (%)</td><td>No-MD Standard housing</td><td>t(15) = 4.30</td><td>.001</td><td>t(15) = 3.62</td><td>.003</td></tr>
<tr><td>No-MD Complex housing</td><td>t(11) = 4.85</td><td>.001</td><td>t(11) = 3.04</td><td>.011</td></tr>
<tr><td>MD Standard housing</td><td>t(15) = 5.85</td><td><.001</td><td>t(15) = 4.08</td><td>.001</td></tr>
<tr><td>MD Complex housing</td><td>t(14) = 1.45</td><td>.168</td><td>t(14) = 5.27</td><td><.001</td></tr>
<tr><td rowspan="4">Social discrimination: Discrimination index (%)</td><td>No-MD Standard housing</td><td>t(15) = 5.43</td><td><.001</td><td>t(15) = -1.88</td><td>.080</td></tr>
<tr><td>No-MD Complex housing</td><td>t(11) = 6.06</td><td><.001</td><td>t(11) = -0.35</td><td>.731</td></tr>
<tr><td>MD Standard housing</td><td>t(15) = 3.23</td><td>.006</td><td>t(15) = 1.22</td><td>.243</td></tr>
<tr><td>MD Complex housing</td><td>t(14) = 1.51</td><td>.153</td><td>t(14) = 0.05</td><td>.964</td></tr>
</table>
